# LncRNA SNHG20 predicts a poor prognosis and promotes cell progression in epithelial ovarian cancer

**DOI:** 10.1042/BSR20182186

**Published:** 2019-04-02

**Authors:** Dandan Wang, Jianrong Dai, Shunyu Hou, Yonghong Qian

**Affiliations:** Department of Gynecology and Obstetrics, The Affiliated Suzhou Hospital of Nanjing Medical University, Suzhou Municipal Hospital, Suzhou, Jiangsu, P.R. China

**Keywords:** cell proliferation, epithelial ovarian cancer, metastasis, prognosis, SNHG20

## Abstract

The long noncoding RNA small nucleolar RNA host gene 20 (SNHG20) has been demonstrated to play a crucial role in cancer progression. However, the functions of SNHG20 in epithelial ovarian cancer (EOC) are not well established. The aim of the present study was to investigate SNHG20 clinical significance and its underlying mechanism in proliferation and metastasis in EOC. The expression level of SNHG20 was identified via *in situ* hybridization (ISH) and quantitative RT-PCR (qRT-PCR). The proliferative and metastatic capacities by silencing SNHG20 expression in A2780 and CAOV-3 cells were measured by cell counting kit-8 (CCK-8) and transwell assays. The molecular mRNA and protein expressions were examined using qRT-PCR, Western blot, and double immunofluorescent staining. SNHG20 expression was markedly higher in serous EOC tissues than that in adjacent tissues and closely correlated with histological grade and lymph node (LN) status. Patients with high SNHG20 showed a shorter overall survival (OS) and SNHG20 was an independent risk factor for the prognosis of serous EOC. Knockdown of SNHG20 remarkably inhibited EOC cell proliferation, migration, and invasion, which was associated with dysregulation of P21, Cyclin D1, E-cadherin, and Vimentin. These results suggest that SNHG20 may serve as an independent prognostic predictor and function as a noncoding oncogene in EOC progression, which might be a possible novel diagnostic marker and treatment target.

## Introduction

Ovarian cancer (OC) is the most lethal gynecological malignancies and the fifth leading cause of death in women, with 22240 new cases and approximately 14070 deaths in 2018 [1]. At present, advanced treatments such as surgical operation, chemotherapy, radiotherapy, and molecular targetted therapy are used for OC, but its overall survival (OS) rate has not improved in essence. Notably, cancer metastasis and postoperative recurrence closely attribute to the poor prognosis of OC patients. Taking these into account, it is necessary to investigate the mechanisms mediating in the progression and development of OC.

Long noncoding RNAs (lncRNAs), a class of longer than 200 nts RNAs and without ORF, play critical roles in cell proliferation, differentiation, chromosome inactivation, metastasis, and autophagy [2–6]. Recently, accumulating shreds of evidence suggested that aberrant expressions of lncRNAs are associated with the progression of various carcinomas including OC [7–11]. Small nucleolar RNA host gene 20 (SNHG20), a novel 2183-nt in length lncRNA located at 17q25.2 on the consistent strand to SEC14-like lipid binding 1 gene, was originally identified in hepatocellular cancer [12]. Subsequently, the oncogene function of SNHG20 was identified in human cancer, such as lung cancer [11], osteosarcoma [13], bladder cancer [14], gastric cancer [15], and colorectal cancer [16]. For example, Guo et al. [17] showed that SNHG20 promoted cell proliferation and invasion via miR-140-5p-ADAM10 axis and MEK/ERK signaling pathway in cervical cancer. He et al. [18] suggested that overexpression of SNHG20 promoted OC progression through Wnt/β-catenin signaling. However, the specific expression profiling and function of SNHG20 in epithelial OC (EOC) still remain largely unclear.

In the current study, we tested the expression level of SNHG20 in serous EOC tissues by *in situ* hybridization (ISH) and quantitative RT-PCR (qRT-PCR) assays, and analyzed its clinical significance. Furthermore, we investigated knockdown of SNHG20 on the development of EOC cells via cell counting kit-8 (CCK-8) and transwell assays. In addition, we identified its potential molecular mechanisms involvement in EOC progression through qRT-PCR, Western blot, and double immunofluorescent staining. In summary, above results suggested that SNHG20, functioning as a tumor oncogene, plays a significant role in the progression of EOC by regulating the proliferation regulators and epithelial–mesenchymal transition (EMT)-related proteins, which indicates that it may serve as a marker contributing to the diagnosis, treatment, and prognosis assessment of EOC.

## Materials and methods

### Patient samples collection

The present study was approved by the Medical Ethics Committee of the Affiliated Suzhou Hospital of Nanjing Medical University. Sixty fresh serous EOC specimens and fifteen adjacent normal tissues were obtained at the Suzhou Municipal Hospital. All fresh samples were immediately snap-frozen in liquid nitrogen at −80°C and made into paraffin-embedded histological sections, respectively. All above patients had complete clinical data including age, histological grade, FIGO stage, lymph node (LN) status, and chemotherapeutic effect (Table 1). The present study was conducted based on the Declaration of Helsinki and all the patients provided written informed consent prior to participation.

**Table 1:** Correlation of the expression of SNHG20 with clinicopathological features

Variable	*n*	SNHG20 expression		Chi-squared test *P*-value
		High	Low	
Ages (years)				
≤55	34	20	14	0.407
>55	26	18	8	
Histological grade				
Well	26	10	16	0.001**
Moderate	18	17	1	
Poor	16	11	5	
FIGO stage				
I+II	26	17	9	0.773
III+IV	34	21	13	
LN status				
Positive	21	19	2	0.001**
Negative	39	19	20	
Chemotherapy				
Resistant	33	22	11	0.554
Sensitive	27	16	11	

**P<0.01.

### Cell culture and transfection

The human EOC cell lines IGROV-1, CAOV-3, A2780, and OVCAR-3 were purchased from Cell Culture Collection of Shanghai. Normal Human Ovarian Surface Epithelial cells (HOSEpiC) were acquired from ScienCell Research Laboratories (Carlsbad). IGROV-1, A2780, OVCAR-3, and HOSEpiC cells were cultured in RPMI-1640 medium supplemented with 10% fetal bovine serum (FBS) (Gibco, U.S.A.) and 1% antibiotics. CAOV-3 cells were maintained in DMEM. All cell lines were maintained at 37°C with a humidified atmosphere of 5% CO_2_ in incubator. SNHG20-specific siRNA and negative control (NC) in the present study were purchased from RiboBio (Guangzhou, China). The siRNA sequences were as shown in Table 3. All those were transfected into cells by Lipofectamine 3000 (Invitrogen) according to the manufacturer’s instructions.

### ISH

RNA ISH for SNHG20 detection was performed using Enhanced Sensitive ISH Detection Kit I (BOSTER, Wuhan, China) in accordance with the manufacturer’s protocol. The ISH intensity of SNHG20 was assessed independently by two researchers using the following system as described previously [12]. The score standard for the staining intensity were defined as 0 (negative), 1 (weak), 2 (moderate), and 3 (strong), respectively. The score of staining extent was scored as follows: ≤10% as 0; 11–25% as 1; 26–50% as 2; 51–75% as 3; and ≥76% as 4. The final scores of SNHG20 were calculated with intensity score + extent score. Total scores of less than 3 were considered the low-expression group.

### RNA isolation, cDNA synthesis, and qRT-PCR

Total RNA from EOC cells and tissues was isolated using TRIzol reagent (Invitrogen, Carlsbad, CA). RNA was reverse transcribed into cDNA with a reverse transcription (RT) reagent kit in a 20-μl reaction volume with 500 ng RNA and qRT-PCR was performed following the manufacturer’s instructions (Takara, Dalian, China). The primers (Invitrogen, Shanghai, China) for analysis were listed in Table 4. The reaction of SNHG20 was performed via the following condition: denaturation at 95°C for 30 s, followed by 40 cycles of 95°C for 5 s, 60°C for 20 s. All qRT-PCR reactions were performed in triplicate. The expression levels were normalized to the content of *β-actin* mRNA and calculated by the 2^−ΔΔ*C*^_t_ method.

### Cell viability assay

Cells transfected with 10 nM si-SNHG20 and NC were seeded in 96-well plates at a density of 5000 cells per well in triplicate. Cell viability was tested by the CCK-8 assay (Dojindo, Kumamoto, Japan) at 0, 24, 48, and 72 h.The optical density (OD) value for each was well recorded at 450 nm on a spectrophotometer (XFluor4 version: V4.51). At least three independent experiments were performed.

### Cell migration and invasion assays

Effect of SNHG20 on cell migration was detected by 24-well transwell plate (6.5 mm) with 8.0-μm pore size polyethylene membrane inserts (Corning, NY, U.S.A.) and transwell membranes were embedded with Matrigel (BD Biosciences, U.S.A.) for cell invasion assay. Cells were seeded in medium without FBS on the top chamber and a volume of 500 μl culture medium with 10% FBS was transferred into the bottom chamber. After 48 h incubation, cells were fixed with 4% paraformaldehyde, stained with Crystal Violet solution, and the non-migration or non-invasion cells were removed with a cotton swab. For both assays, cells were counted under a microscope in five randomly selected fields per well. All experiments were independently performed three times.

### Western blot

Whole-cell lysates were prepared, quantitated, and then protein was separated by 10% SDS/PAGE gel (Beyotime, China). After electrophoresis, protein was transferred on to PVDF membranes (Millipore, Boston, U.S.A.) and then blocked in 5% BSA. At 4°C overnight, the membranes were incubated with primary antibodies, including P21 (1:1000, Abcam, ab109520), Cyclin D1 (1:1000, Santa Cruz Biotechnology, sc-450), E-cadherin (1:1000, BD, 610181), Vimentin (1:1000, Abcam, ab92547), and β-actin (1:5000, ProteinTech, 66009-1-Ig). After incubating with secondary antibody, protein signals were detected by enhanced chemiluminescence (ECL).

### Double immunofluorescent staining

The EOC cells transfected with si-SNHG20 and si-NC were simultaneously incubated with primary antibodies against P21 (1:50, Abcam, ab109520) and Cyclin D1 (1:50, Santa Cruz Biotechnology, sc-450), or E-cadherin (1:75, BD, 610181) and Vimentin (1:50, Abcam, ab92547). Rabbit or mouse IgG replaced the primary antibody for NC. The effect concentration of tetraethyl rhodamine isothiocyanate (TRITC) and fluorescein isothiocyanate (FITC) was 1:50. 4′,6-diamidino-2-phenylindole (DAPI) counterstained nuclei. At least three independent experiments were done.

### Statistical analysis

SPSS software version 17.0 (IBM SPSS, Chicago, IL) was used for statistical analysis. χ^2^, variance analysis, and *t* test were used to evaluate the statistical relevance between different groups. Kaplan–Meier and log-rank methods were used to assess and examine the survival curves. Cox regression model was used to analyze the risk factors. A value of *P*<0.05 was considered statistically significant.

## Results

### SNHG20 was up-regulated in serous EOC tissues and predicted a poor prognosis

**Figure 1:**
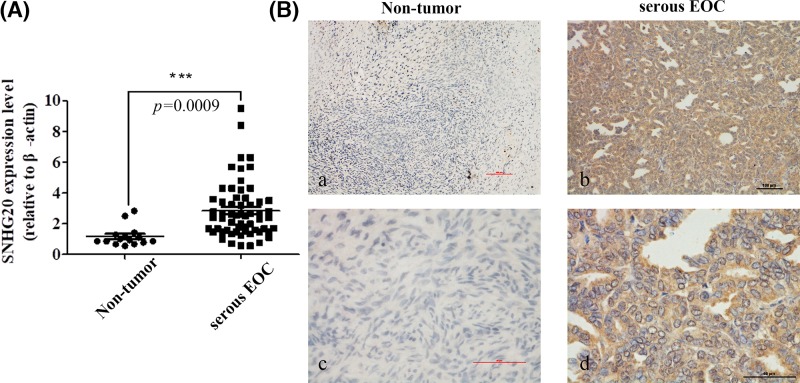
The expression of SNHG20 in serous EOC and non-tumor tissues were examined via qRT-PCR and ISH assays (**A**) Up-regulation of SNHG20 was observed in serous EOC specimens (*n*=60) compared with non-tumor counterparts identified by qRT-PCR (*n*=15). (**B**) Representative images of SNHG20 in serous EOC specimens (**b**,**d**) and non-tumor counterparts (**a**,**c**) (magnification: a,b 100×; c,d 400×).

**Figure 2:**
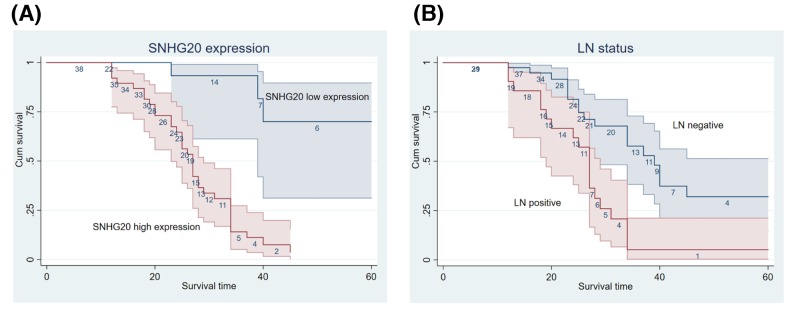
Kaplan–Meier analysis of serous EOC Patients with high SNHG20 expression (**A**) and LN positive (**B**) showed shorter OS.

In the present study, we identified SNHG20 expression in human serous EOC tissues by qRT-PCR and found that its level was overexpressed in serous EOC tissues compared with adjacent histologically normal tissues (Figure 1A). To further validate dysregulated expression of SNHG20, it was determined in 60 serous EOC samples and 15 adjacent normal tissues using ISH (Figure 1B). The result was consistent with qRT-PCR data; 38 out of 60 (63.3%) samples were grouped as high SNHG20 expression using a cutoff score ≥ 3. Clinicopathological characteristics of the 60 serous EOC patients were summarized in Table 1. Statistical analysis indicated that SNHG20 overexpression was positively correlated with poor histological grade and LN metastasis (*P*=0.001). However, other features including ages (*P*=0.407), FIGO stage (*P*=0.773), and chemotherapy effects (*P*=0.554) showed no significant association with SNHG20 expression. Kaplan–Meier analysis showed that SNHG20 overexpression and LN metastasis were significantly associated with a shortened OS, predicting a poor prognosis (*P*<0.001) (Figure 2 and Table 2). Further, SNHG20 expression and LN status were included in the multivariate Cox regression model and statistical analysis showed that high expression of SNHG20 was an independent risk factor for the prognosis of serous EOC (Table 2).

**Table 2:** Survival analysis and prognosis analysis of serous EOC

Variable	Characteristics	(Log-rank) *P*-value
Age (years)	≤55 vs. >55	0.989
Histological grade	Well vs. Moderate and Poor	0.071
FIGO stage	I+II vs. III+IV	0.763
LN status	Positive vs. Negative	<0.001
Chemotherapy	Resistant vs. Sensitive	0.920
SNHG20	High vs. Low	<0.001
Multivariate Cox regression analysis of patients with EOC		
Variable	*P*-value	Hazard ratio (95% CI)
LN	0.044	0.483 (0.238–0.981)
SNHG20	<0.001	0.109 (0.032–0.369)

### Modulation of SNHG20 expression in EOC cells

**Figure 3:**
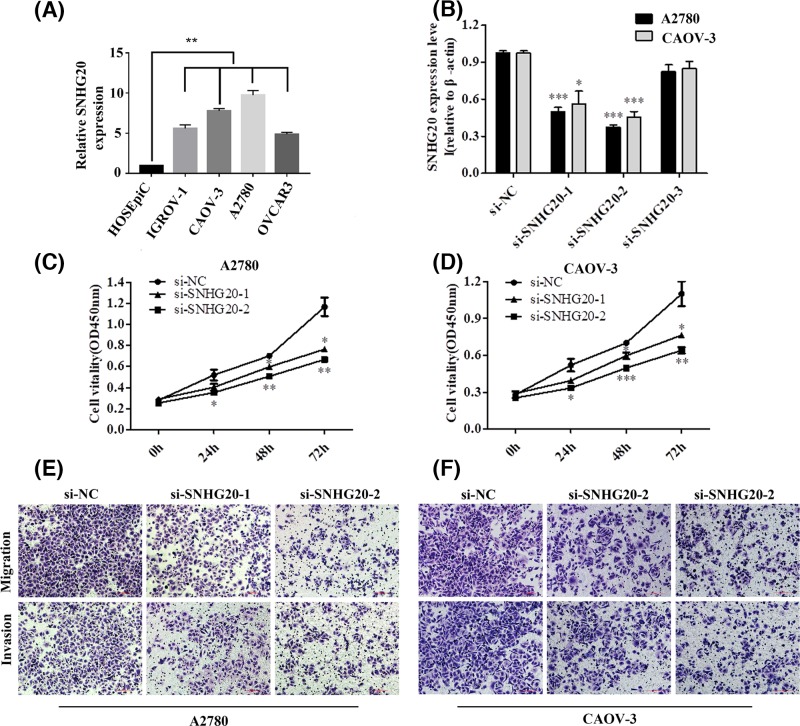
SNHG20 knockdown inhibited EOC cells proliferation, migration, and invasion (**A**) qRT-PCR analysis of SNHG20 expression in HOSEpiC and EOC cell lines (**P<0.01). (**B**) The efficiency of siRNAs targetting at SNHG20 was detected via qRT-PCR in A2780 and CAOV-3 (*P<0.05, ***P<0.001). (**C**,**D**) Suppression of SNHG20 reduced EOC cells proliferation, as determined by CCK-8 assay (*P<0.05, **P<0.01, and ***P<0.001). (**E**,**F**) Knockdown of SNHG20 in EOC cells inhibited their migrative and invasive abilities (magnification 200×).

To assess the biological function of SNHG20 in EOC progression, we examined its expression level in a range of EOC cell lines using qRT-PCR. As shown in Figure 3A, SNHG20 expression was up-regulated in EOC cell lines, especially in A2780 and CAOV-3 compared with that in HOSEpiC. Then we silenced the expression of SNHG20 in above two cell lines via siRNAs. Three SNHG20-specific siRNAs sequence were listed in Table 3. qRT-PCR analysis was performed at 48 h post-transfection and indicated that si-SNHG20-1 and -2 effectively inhibited SNHG20 expression (Figure 3B). Therefore, these two siRNAs were used in the following research.

**Table 3:** The sequences of siRNAs targetting SNHG20

Name	Sense	Antisense
si-SNHG20-1	GCCUAGGAUCAUCCAGGUUTT	AACCUGGAUGAUCCUAGGCTT
si-SNHG20-2	CCUGUUGUGUAUGGCUAUATT	UAUAGCCAUACACAACAGGTT
si-SNHG20-3	GCCACUCACAAGAGUGUAUTT	AUACACUCUUGUGAGUGGCTT

**Table 4:** The primers for qRT-PCR

Gene	Sense primer	Antisense primer
*SNHG20*	ATGGCTATAAATAGATACACGC	GGTACAAACAGGGAGGGA
*P21*	GTCAGAACCGGCTGGGGATG	CTCCTCCCAACTC ATCCCGG
*Cyclin D1*	CCTCGGTGTCCTACTTCAAA	GGGATGGTCTCCTTCATCTT
*E-cadherin*	TGCCTGAGAACGAGGCTAAC	TGGGGGCTTCATTCACATCC
*Vimentin*	AACTTAGGGGCGCTCTTGTC	CCTGCTGTCCCGCCG
*β-actin*	TCAAGATCATTGCTCCTCCTGA	CTCGTCATACTCCTGCTTGCTG

### Knockdown of SNHG20 inhibited EOC cells proliferation, migration, and invasion

First, we investigated the effect of SNHG20 knockdown on cell proliferation. The results of CCK-8 assay showed that A2780 and CAOV-3 transfected with si-SNHG20-1 or -2 impaired cell growth (Figure 3C,D). In addition, inhibition of SNHG20 decreased migration and invasion abilities in above two EOC cell lines as identified by transwell assays (Figure 3E,F). In summary, these data indicated that down-regulation of SNHG20 markedly inhibited EOC cell proliferation and motility.

### SNHG20 regulated proliferation regulators and EMT-associated genes expression in EOC cells

**Figure 4:**
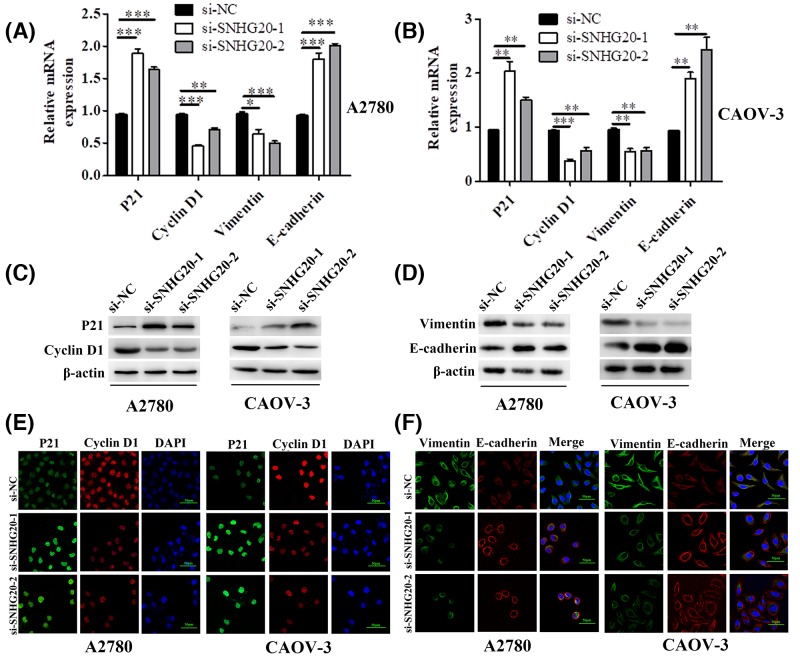
SNHG20 knockdown regulated several genes related to proliferation and metastasis (**A**,**B**) The mRNA level of P21, Cyclin D1, Vimentin, and E-cadherin was detected by qRT-PCR in A2780 and CAOV-3 cells transfecting with si-SNHG20-1 or -2 (*P<0.05, **P<0.01, and ***P<0.001). (**C**–**F**) The protein level of P21, Cyclin D1, Vimentin, and E-cadherin was detected by Western blot and immunofluorescence in above EOC cells (magnification 400×).

In order to explore the molecular mechanism of SNHG20 in regulating EOC cell proliferation and motility, we performed qRT-PCR to detect the expression level of proliferation regulators and EMT-associated genes including *P21, Cyclin D1, E-cadherin*, and *Vimentin*. As shown in Figure 4A,B, we found that the mRNAs of P21 and E-cadherin were significantly increased, while Cyclin D1 and Vimentin were down-regulated at post-transfection with SNHG20 siRNA-1 and -2. In addition, Western blot results indicated that the protein levels showed the same trend as shown in levels of mRNA (Figure 4C,D). We further conducted double immunofluorescent staining to confirm above genes protein expression and explicit their cell localization. The results revealed that P21 and Cyclin D1 coloration mainly occurred in cell nucleus, E-cadherin localized to cell–cell boundaries, and Vimentin coloration mainly localized in cell membrane and cytoplasm (Figure 4E,F). Moreover, the trend of their protein expressions was the same as the results of Western blot. These data suggested that SNHG20 promoted EOC cell proliferation, migration, and invasion partly through the regulation of P21, Cyclin D1, E-cadherin, and Vimentin.

## Discussion

OC is one of the most common gynecologic cancers worldwide. Although the treatment strategies have improved significantly in recent years, its 5-year survival rate is approximately 25–45% due to tumor recurrence, metastasis, chemoresistance, and immune escape. Survival analysis confirms that metastasis is an important risk factor for poor prognosis. Hence, to clarify the mechanisms of migration and invasion could be of great value in OC treatment.

LncRNAs, mainly being members of the noncoding RNA family, have been identified to participate in many cellular processes and their dysregulations seem to correlate with the progression and development of different diseases. Newly confirmed and functional lncRNA, SNHG20 influences not only cancer cells proliferation, metastasis and as prognostic factors, but also EMT and mitochondrial apoptosis [13,19,20]. In 2018, He et al. [18] found that SNHG20 was overexpressed in OC and promoted cell progression via Wnt/β-catenin signaling pathway. To date, the investigations of SNHG20 in EOC were limited.

In the present paper, first we detected SNHG20 expression level in serous EOC tissues and then explored the association of highly expressed SNHG20 with clinicopathological features and prognosis. We found that SNHG20 was markedly up-regulated in serous EOC tissues. Overexpression of SNHG20 closely associated with poor histological grade and LN metastasis, and it might be regarded as an independent risk factor for prognosis. Recently, the prognostic value of SNHG20 has been suggested in a series of cancers. Guan et al. found that patients with high SNHG20 had shorter OS and poorer progression-free survival (PFS) in non-small cell lung cancer (NSCLC) [11]. Zhang et al. [12] observed that SNHG20 up-regulation was an independent prognostic factor in hepatocellular carcinoma. Similar results about the prognostic significance of SNHG20 in colorectal cancer were reported by Li et al. [16]. In addition, Guo et al. [17] pointed out that highly expressed SNHG20 was associated with unfavorable OS in patients with cervical cancer. To gain insight into the biological function of SNHG20 in EOC, loss-of-function was performed in cell lines. We found that SNHG20 knockdown could inhibit cell proliferation, migration, and invasion. These data suggested that SNHG20 might act as an oncogene in the tumorigenesis of EOC.

Cyclin D1 leads to dysregulated CDK activity, cell growth under conditions of restricted mitogenic signaling, bypass of key cellular checkpoints, and ultimately, neoplastic growth [21]. Cell proliferation is regulated by numerous cell processes, such as cell cycle and apoptosis, which is a complex cell program. P21, also known as p21WAF1/CIP1, is accepted as a potent CDK inhibitor (CKI). It physically interacts with a series of cyclins, hence functioning as a regulator of cell cycle progression and further mediating cell proliferation [22]. Metastasis is a characteristic of malignant cancer which is the leading cause of poor prognosis. In recent years, research findings suggest that EMT plays a major role in tumor metastasis, which may regulate mesothelial cell clearance and drive early dissemination into the peritoneal cavity [23].Moreover, EMT is a biologic process where cells lose their epithelial marker such as E-cadherin and obtain mesenchymal-like phenotype marker including Vimentin, which usually occurs in the initial stage of tumor metastasis.

To ascertain the possible mechanism for SNHG20 mediating in EOC proliferation and motility, we detected several proliferation regulators and EMT-associated genes expression. Using qRT-PCR and Western blot, our study showed that P21 and E-cadherin were significantly increased, while Cyclin D1 and Vimentin were down-regulated at post-transfection with SNHG20 siRNA-1 and -2. In addition, we conducted double immunofluorescent staining and results revealed that P21 and Cyclin D1 mainly occurred in cell nucleus, E-cadherin localized to cell-cell boundaries, and Vimentin coloration localized in cell membrane and cytoplasm. Moreover, the trend of above protein expression levels was the same as the results of Western blot.

Present studies have identified that silenced SNHG20 inhibited the proliferation, invasion and induced apoptosis in osteosarcoma via miR-139/RUNX2 axis [13]. Guo et al. [17] found that LncRNA SNHG20 promoted cervical cancer progression through miR-140-5p-ADAM10. Meanwhile, Chen et al. [11] pointed out that SNHG20 functioned as an oncogene partly via interacting with EZH2 and repressing P21 in NSCLC cells. According to these studies, the most common way in which SNHG20 functions is acting as a sponge for miRNAs or integrating with proteins or mRNAs to regulate downstream target genes. Our results of ISH assay indicated that SNHG20 mainly located in cell cytoplasm and we speculated that it may play a role in EOC progression through above two means. This will be further researched in future studies.

In summary, our findings showed that SNHG20 was markedly overexpressed in serous EOC tissues and highly expressed SNHG20 was closely associated with poor histological grade, LN metastasis and poor prognosis, which might be an independent risk factor for the prognosis of serous EOC. Silenced SNHG20 inhibited cell proliferation, migration, and invasion partly via the regulation of Cyclin D1, P21, E-cadherin, and Vimentin. This current research provides a new perspective for SNHG20 functioned as a noncoding oncogene in EOC progression and it could be a possible novel diagnostic marker and treatment target. However, the detailed potential mechanisms by which SNHG20 involves in EOC cells progression remain to be further understood.

## Abbreviations

CCK-8: cell counting kit-8
EMT: epithelial–mesenchymal transition
EOC: epithelial ovarian cancer
FBS: fetal bovine serum
HOSEpiC: human ovarian surface epithelial cell
ISH: *in situ* hybridization
LN: lymph node
IncRna: long noncoding RNA
NC: negative control
NSCLC: non-small cell lung cancer
OC: ovarian cancer
OS: overall survival
qRT-PCR: quantitative RT-PCR
SNHG20: small nucleolar RNA host gene 20
